# Soft Matter: A Tale of Eco-Friendly Materials, Self-Organised Phases and Biological Impact

**DOI:** 10.3390/ma19112316

**Published:** 2026-05-31

**Authors:** Ingo Dierking

**Affiliations:** Department of Physics and Astronomy, University of Manchester, Oxford Road, Manchester M13 9PL, UK; ingo.dierking@manchester.ac.uk

The field of soft matter [1,2,3,4] is rather broad and diverse, covering not only the classic areas of polymers [5,6,7], colloids [8,9,10,11,12,13] and liquid crystals [14,15,16,17,18], but also foams [19], granular matter [20], surfactants [21,22,23], active matter [24,25,26], biological tissue [27], membranes, gels [28,29,30] and complex fluids [31,32]. It encompasses organic, inorganic and biological building blocks, which form structured phases via self-assembly. Such materials have very small elastic constants, and their predominant physical behaviour occurs at energies comparable to the thermal energy kT at room temperature. The family of building blocks is widespread, ranging from nanoparticles, nanotubes, 2D materials and composites to macromolecules, surfactant micelles, vesicles, dendrimers and lipids, all the way to peptides, proteins and DNA. The materials are widespread; they have practical applications in the food [33,34,35] and pharmaceutical [36] industries, as high-tech and smart building materials in robotics and sensors [37,38], biomaterials in healthcare [39], and cosmetics [40], and are also found in nanotechnology [41,42], photonics [43,44], sensors [45] and electronics [46,47].

The three papers, selected as Editor’s Choice papers, signify the broadness and impact of the field of soft matter.

The first paper [48] is a study of deep eutectic solvents as an emerging replacement of organic solvents, being low cost, environmentally friendly, and non-flammable. The investigation is carried out via a range of modern nuclear magnetic resonant methods, namely NMR dispersion, providing molecular level information, and Pulsed Field Gradient NMR, revealing composition dependent properties. The deep eutectic solvent investigated is a system of a metal salt, lithium bis(trifluoromethanesulfonyl)imide with hydrogen bond donors, and pyrazole at varying LiTFSI:PYR composition ratios.

There are current examples of compositions that exhibit low self-diffusion coefficients, high viscosity, and an inverse relationship between the latter and ionic conductivity. This is a step on the path towards a replacement of organic solvents in batteries, and a broader, fundamental understanding of the related molecular processes. Consequently, it is also a step toward the use of lithium-ion batteries on a larger scale for handheld devices, wireless technologies, electric transportation, and the storage of renewable energy with eco-friendly technology via reduction in the use of organic solvents.

The next article [49] is related to a typical classical soft matter material: liquid crystals. For nearly a century, the holy grail of liquid crystal research has been the discovery of ferroelectricity in nematic materials. Nematics have been known since the beginning of liquid crystal research and have first found their application around 50 years ago in the displays of pocket calculators and wrist watches; this later developed into their use in flat panel screens of laptops, mobile phones, and TVs. Ferroelectric (smectic) liquid crystals have been known for a similar time period, since 1975, yet ferroelectric nematics offer the potential production of devices with standard technologies and low viscosity, in combination with fast switching and very low power consumption. Such materials were only discovered a few years ago. In the paper, a reference ferroelectric nematic material is discussed which is industry made, stable, and most importantly, acts as a ferroelectric nematic at room temperature, the key ingredient for device applications. Reported in this paper is a comprehensive characterisation of structural, optical, dielectric and elastic properties across all phases, including the temperature dependence of the ferroelectric polarisation, the viscosity, threshold voltage and response time. The compound is then compared to a previous “standard” material which was lab synthesised and not a room temperature liquid crystal. The phase sequence of the ferroelectric nematic, in contrast to the previously known ferroelectric liquid crystals that are related to chirality, is as follows: paraelectric nematic–antiferroelectric nematic–ferroelectric nematic; this is in line with solid state materials.

Hyaluronic acid has a unique exposure in bio-composite gels due to its water binding properties which allows the swelling of the gels, a property which is exploited in the food and cosmetics industry in addition to the areas of pharmaceutics and drug delivery. In the third paper, the effect of different concentrations of hyaluronic acid on the physical properties, such as mechanical and rheological behaviour, texture and colour, of furcellaran-based composite bio-gels and emulsion-gels was investigated [50]. Here, the antioxidant potential was also studied, which was found to strongly increase with hyaluronic acid concentration; this signifies the outstanding potential of these materials as delivery systems for hydrophilic and lipophilic bioactive materials.

These three papers reflect the multi-disciplinarity and breadth of soft matter research, ranging in this case from deep eutectic solvents to liquid crystals and on to bio-composite gels. Going beyond fundamental research, in terms of application potential, the materials promise developments in environmentally friendly battery production, regenerative energy storage, electrooptic and sensor materials, and biological materials. We aim for these articles to reach a broad readership, of which they are well deserved.

As made clear from these Editor’s Choice publications, the field of soft matter is not only a highly interdisciplinary area of science, but is in fact a rather multidisciplinary one. Many studies combine not only experimental, theoretical and simulation physics with synthetic, physicochemical and analytical chemistry, but also characterisation and applicational materials science, and sometimes even mathematics and electrical engineering. More recently, biological, biomaterials and biomedical aspects are increasingly reliant on methodologies taken from soft condensed matter. This multi-disciplinarity has paved the way to novel future directions, such as switchable photonics and micro-photonics [51], drug delivery and biotechnology [52], active, living and topological matter [53], soft matter electronics [54], and soft robotics [55,56], just to name a few. It is thus reasonable to assume that in the coming years, soft matter science and research will be devoted to the investigation of systems that will bridge the gap between more conventional soft matter fields like polymers, colloids, gels, or liquid crystals and cell biology and other nature-mimicking materials in the fields of photonics, medical applications, sensors and biosensors, in addition to more fundamental topics like topology, solitons and knot theory. In any case, this will constitute an exciting endeavour into novel materials and applications.
